# The Prevalence of Cardiovascular Complications and Causes of AngioJet Failure: A Post-Marketing Surveillance Study Based on the MAUDE (Manufacturer and User Facility Device Experience) Database

**DOI:** 10.7759/cureus.42824

**Published:** 2023-08-01

**Authors:** Chaitu Dandu, Dhruvil Patel, Ryan Naughton, Neel N Patel, Bandar Alyami, Maria Najam, Mustafa Bdiwi, Rashid Alhusain, Yasar Sattar, M. Chadi Alraies

**Affiliations:** 1 Vascular Surgery, Wayne State University School of Medicine, Detroit, USA; 2 Internal Medicine, Wayne State University School of Medicine, Detroit, USA; 3 Internal Medicine, Wayne State University Detroit Medical Center, Detroit, USA; 4 Anesthesiology, University of Miami Miller School of Medicine, Jackson Memorial Hospital, Miami, USA; 5 Internal Medicine, New York Medical College/Landmark Medical Center, Woonsocket, USA; 6 Graduate Medical Education, B J Medical College, Ahmedabad, IND; 7 Medicine, West Virginia University School of Medicine, Morgantown, USA; 8 Internal Medicine, Knapp Medical Center, University of Texas Rio Grand Valley, Weslaco, USA; 9 Internal Medicine, Detroit Medical Center, Detroit, USA; 10 Internal Medicine, Icahn School of Medicine at Mount Sinai, New York, USA; 11 Cardiology, Detroit Medical Center, Detroit, USA

**Keywords:** post-marketing surveillance, peripheral arterial diseases, aspiration thrombectomy, maude, angiojet

## Abstract

Background and objective

Aspiration thrombectomy devices, such as the AngioJet Solent Omni (Boston Scientific Corporation, Marlborough, MA) have been approved by the US FDA for the treatment of thrombi in peripheral arterial disease, venous disease, and AV fistulas. However, there is a dearth of real-world data on the most common modes of failure and complications associated with the AngioJet Solent Omni. In this study, we aimed to address this scarcity of data.

Methods

The MAUDE (Manufacturer and User Facility Device Experience) database was queried for reports of device failure and adverse events spanning the period from October 2012 to December 2021.

Results

A total of 499 events were reported during the study period. After the exclusion of duplicate reports, the final analysis included 450 reports. The most common mode of failure was catheter breakage/kinking during suction thrombectomy with 137 reports (30%). The most common vessel associated with events was the superficial femoral artery or vein, which was documented in 82 reports (18.2%). The most common adverse clinical outcome was the embedding of a piece of the device in the patient, which occurred in seven reports (1.6%). There were seven (1.6%) events of death reported during the period studied.

Conclusions

Based on our findings, theAngioJet Solent Omni device provides promising results; however, it is important to evaluate device safety. It is associated with complications including device embedment, catheter breakage/kinking, and death, and these adverse events are linked to patient characteristics and risk factors.

## Introduction

Acute limb ischemia (ALI), typically secondary to thrombotic or embolic disease, is a severe limb-threatening disease with potentially lethal sequelae. Without prompt recognition and treatment, ALI can often lead to long-term disability, severe quality of life degradation, and increased economic burden [1]. Thanks to advancements in medical technology and techniques, many mechanical thrombectomy devices have been introduced to treat ALI with minimally invasive interventions. These devices cut across a wide spectrum of approaches and include percutaneous mechanical thrombolysis, rheolytic thrombectomy, rotational thrombectomy, thrombus fragmentation, and aspirational techniques [2-4].

The AngioJet Solent Omni (Boston Scientific Corporation, Marlborough, MA) is one such device. It is a rheolytic mechanical thrombectomy device used in small and large arterial vessel thrombolysis. The device has been widely reported to have numerous advantages over surgical methods in scenarios such as inferior vena cava filter-related thrombosis and hemodialysis graft thrombosis [5-7]. Rheolytic thrombectomy techniques, specifically the AngioJet percutaneous mechanical thrombectomy system, initially create a fluid suction by introducing high-pressure saline and pro-thrombotic medication through catheter openings around the target, which produces a subsequent inwards flow, aspirating the resultant debris as the devices move proximally in the clot [8]. The thrombus is subsequently fragmented by jets as it enters the catheter, which introduces thrombolytics [9-11]. The AngioJet thrombectomy portfolio is used across the spectrum of embolic and thrombotic events, being implemented in large arteries and targeting coronary occlusions, with respective systems designed for each use case that has a different working length [12]. The current AngioJet Solent portfolio consists of the AngioJet Solent Proxi, the AngioJet Solent Omni, and the AngioJet Solent Distal. 

Despite its well-documented advantages, data on real-world failure modes and complications associated with the Angiojet Solent Omni catheter remain scarce. To gain insight into these issues, we analyzed reports pertaining to the Angiojet Solent Omni system in the MAUDE (Manufacturer and User Facility Device Experience) database. The MAUDE database is an FDA-mandated post-market surveillance repository of approved medical devices in the United States that covers a 10-year period [13]. We believe our findings on AngioJet-related reports will help better inform operators about the potential complications associated with this device and thereby encourage them to consider techniques to avoid them during use.

## Materials and methods

Data source

The FDA created the MAUDE database to list adverse events caused by approved medical devices. The MAUDE database consists of reports submitted to the FDA by mandatory reporters (manufacturers, importers, and device user facilities) and voluntary reporters such as healthcare professionals, patients, and consumers. It is publicly available online and de-identified. Therefore, no institutional review board approval was required for this study. We queried the database for reports from January 2012 to December 2021 by using the keyword “AngioJet Solent Omni.”

Outcomes and statistical analysis

The primary outcome measure of this study was the failure modes of the AngioJet Solent Omni. Secondary outcomes included major complications associated with device failure. Target vessels and their relationship with failure outcomes were also analyzed. The MAUDE database cannot capture the overall utilization of the AngioJet Solent Omni in the United States; therefore, the actual incidence rate of each failure or complication type cannot be assessed. Categorical variables were presented as numbers; all statistical analyses were performed using IBM SPSS Statistics V.27.0 (IBM Corp., Armonk, NY).

## Results

Our search elicited a total of 499 reports during the study period from January 2012 to October 2021. After excluding duplicate reports (n=49), 450 reports were included in the final analysis.

The most common target site of interventions implicated in reports was the iliac veins (n=32, 7.11%), followed by iliac arteries (n=16, 3.56%), common femoral vein (n=14, 3.11%), and AV grafts/fistulas (n=10, 2.22%). Vessels in the other category (n=23, 5.11%) included but were not limited to other below-the-knee vessels, subclavian arteries, brachial arteries, mesenteric vessels, and internal carotid arteries. 

**Figure 1:**
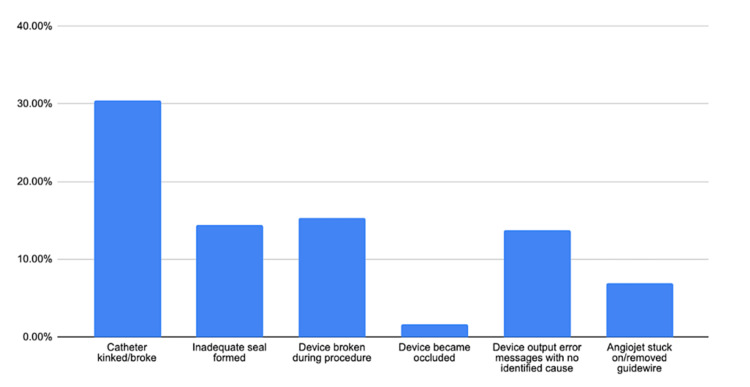
Modes of failure of AngioJet

The most common AngioJet-associated adverse event was the embedment of the device (n=7, 1.60%) followed by arrhythmias (n=3, 0.7%) and myocardial infarction (n=2, 0.4%). Other unspecified adverse events were reported in seven (1.6%) cases. No vessel perforation, dissection, or hematomas were reported as complications of AngioJet use. Most patients suffered no consequences (n=400, 88.9%) from device failure; 12 (2.67%) patients experienced some form of injury but duly recovered. Seven (1.6%) deaths were reported, with no specified etiology (Figure 1).

**Figure 2:**
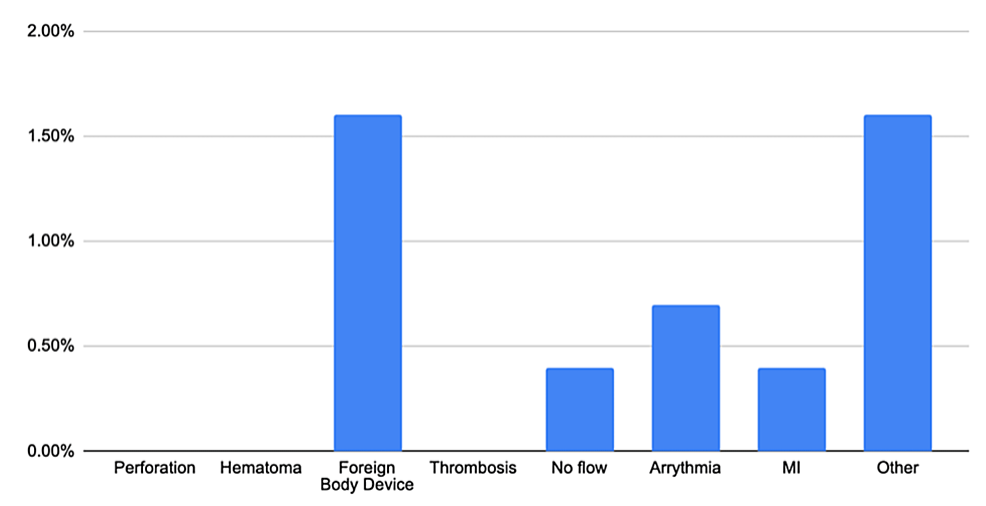
Adverse events associated with AngioJet

The most common pre-procedural mode of failure of AngioJet was breakage of device tubing (n=42, 9.3%), followed by breakage of the catheter device (n=37, 8.3%). Another notable failure was an output error without identifiable cause immediately after the device was hooked to the machine (n=36, 8%) (Figure 2).

The most common modes of failure during the procedure included catheter kinking or breakage (n=137, 30.4%), followed by device breakage (n=69, 15.3%), inadequate seal formation between locks leading to device breakage (n=65, 14.4%), device output error messages with no identified cause (n=62, 13.8%), and AngioJet getting stuck on/removing guidewire (n=31, 6.9%). The findings are summarized in Table 1.

**Table 1:** Summary of the findings

Findings	Values
Total number of events	450
Target vessel	N (%)
AV graft/fistula	10 (2.22%)
Pulmonary artery	3 (0.67%)
Illiac artery	16 (3.56%)
Common femoral artery	9 (2.00%)
Superficial femoral artery	8 (1.78%)
Popliteal artery	6 (1.33%)
Anterior tibial artery	2 (0.44%)
Arterial bypass with vein/graft	6 (1.33%)
IVC	8 (1.78%)
Illiac vein	32 (7.11%)
Common femoral vein	14 (3.11%)
Superficial femoral vein	8 (1.78%)
Popliteal vein	8 (1.78%)
Other	23 (5.11%)
Unknown	260 (57.78%)
Pre-procedure modes of failure	N (%)
Cather tip contaminated	3 (0.7%)
Catheter device broken	37 (8.2%)
Tubing of the device broken	42 (9.3%)
Pump bay of the device broken	3 (0.7%)
Device was hooked to the machine and output error of unknown status with no identifiable cause	36 (8.0%)
In-procedure modes of failure	N (%)
Catheter kinked/broken	137 (30.4%)
Inadequate seal formed	65 (14.4%)
Device broken during the procedure	69 (15.3%)
Device became occluded	7 (1.6%)
Device output error messages with no identified cause	62 (13.8%)
AngioJet stuck on/removed guidewire	31 (6.9%)
Clinical adverse events	N (%)
Perforation	0 (0.0%)
Hematoma	0 (0.0%)
Foreign body device entrapment	13 (1.60%)
Thrombosis	0 (0.0%)
No flow	2 (0.4%)
Arrhythmia	3 (0.67%)
MI	2 (0.4%)
Other	7 (1.60%)
Patient outcomes	N (%)
Death	7 (1.60%)
No consequences	400 (88.89%)
Recovered	12 (2.67%)
Unknown/insufficient information	31 (6.9%)

## Discussion

Based on our analysis of the MAUDE database, the important complications that operators must be aware of when using the device are described as follows: (1) both the breakage of the device tubing and the device catheter were the most common modes of failure prior to device implementation; (2) catheter kinking or breakage constituted the highest proportion of in-procedure failures followed by inadequate seal formation.

To our knowledge, and based on the review of the literature, this is the first study to report specific peri-procedural failure modes of the AngioJet Solent Omni system. We found that both the device catheter kinking or breaking, and device tubing damage were the most common adverse events associated with this device. There are currently no reports of isolated cases that describe these events and their subsequent management in the clinical setting, but both damages of the catheter and/or device tubing in other classes of devices are risk factors for severe complications. Catheter kinking can lead to entrapment, thereby increasing the risk of complications. This phenomenon is seen more commonly in small-vessel interventions. While AngioJet is more frequently used in larger vessels, operators should still be aware of such risks [14,15]. A majority of the reports described no consequences to the patient (n=400, 88.9%); 2.67% (n=12) of patients suffered some form of injury, from which they duly recovered. The findings of these reports necessitate that operators remain aware of the risks involved with using the device.

The most common single-vessel targeted site of arterial intervention was the iliac artery. Arterial thrombosis emerges from preexisting arterial plaque buildup that exerts reactive changes, promoting endothelial damage. Continued insult or rapid destruction of the plaque destroys endothelial integrity and induces platelet activation, which rapidly produces a pro-thrombogenic collection [16]. Larger arteries of the body are increasingly sensitive to plaque collection due to the high pressure and large volume moving through them. The superficial femoral artery is a well-characterized source of peripheral arterial disease with increased plaque burden being linked to worse functional performance. This makes it a prime source for thrombotic pathology [17]. Our data shows that the AngioJet system commonly experiences complications with device use when intervening in the superficial femoral artery. Due to this preponderance for atherosclerotic disease and the importance of managing peripheral artery disease, operators should be aware of the various modes of AngioJet device failure discussed herein to remain vigilant during procedures.

In our review of the intervention reports, there was no vessel damage attributed to AngioJet device implementation. This included events of vessel perforation, dissection, or hematoma. The lack of these events is likely due to the AngioJet mechanism of clot dissolution. Rheolytic thrombectomy avoids direct contact of fragmentation mechanisms with any part of the vessel lumen - due to the source of aspiration jet stream being covered by the device catheter. Although minimal, reports of hematoma and non-occlusive vessel dissection have been documented, usually at catheter introduction sites, and operators must be mindful of such complications [18]. Furthermore, device introduction or unsuccessful evacuation of the entire thrombus can both cause reactive embolism and local infarction, but these events still remain rare and their causes are not well characterized [19,20]. High-risk populations do exist. Specifically, complications from AngioJet device use are well known among patients experiencing a blockage of their arteriovenous fistula - where AngioJet implementation can cause hematoma and venous rupture as reported in a meta-analysis by Chan and Goh, highlighting the importance of operator vigilance in these patients [21]. 

Furthermore, despite the lack of mechanical interaction with vessel walls, the high-pressure saline jet and resultant aspiration forces produced from the AngioJet rheolytic atherectomy cause shearing of red blood cells. This produces a well-known post-procedural event from AngioJet implementation: hemolysis manifesting as hemoglobinuria and subsequent acute kidney injury that can result in acute renal failure - seen consistently as one of if not the most significant complication burden among cohorts undergoing AngioJet implementation [22-24]. Bleeding complications due to device introduction were not reported in our database, but this is another known complication of device introduction, notably both access-site bleeding and gastrointestinal bleed [18,22].

There are a few limitations to this study. Due to the retrospective nature and the voluntary reporting of adverse events by health professionals, selection bias and underreporting are likely. Using the data, operators can only learn about potential risks and complications associated with the device. More prospective studies are needed to further explore the AngioJet system in terms of its safety and efficacy.

## Conclusions

Despite clinical trials demonstrating the safety of the AngioJet catheters, complications can still occur. We hope our findings will serve to better inform operators about potential risks and complications associated with the use of the device. Physicians should be well-trained to use AngioJet catheters and avoid over-torquing, a technique that may lead to catheter breakage and detachment.
